# Giant hydronephrosis presented as a huge abdominal mass in a 16-year-old female: a case report

**DOI:** 10.11604/pamj.2022.41.295.33135

**Published:** 2022-04-12

**Authors:** Waleed Aljbri, Faisal Ahmed, Saif Ghabisha, Menawar Dajenah, Ebrahim Al-shami, Qasem Alyhari, Fawaz Mohammed

**Affiliations:** 1 Department of Urology, School of Medicine, 21 September University, Sana'a, Yemen,; 2 Urology Research Center, Al-Thora General Hospital, Department of Urology, School of Medicine, Ibb University of Medical Sciences, Ibb, Yemen,; 3 Department of General Surgery, School of Medicine, Ibb University of Medical Science, Ibb, Yemen,; 4 Department of Orthopedy, School of Medicine, Ibb University of Medical Sciences, Ibb, Yemen

**Keywords:** Abdominal mass, hydronephrosis, ureteropelvic junction obstruction, case report

## Abstract

Giant hydronephrosis owing to ureteropelvic junction obstruction is a rare condition characterized by the accumulation of more than 1000 ml of urine in the pyelocaliceal system. It could mimic the other benign cystic kidney disease or other causes of abdominal mass in radiologic images. We reported a 16-year-old female who presented with three months of abdominal pain and gradual abdominal mass ingrowth. Abdominal computed tomography scan showed a giant left cystic mass favored hydronephrosis secondary to ureteropelvic junction obstruction. The patient underwent a left nephrectomy, and more than 12 litters of turbid urine were suctioned from the affected kidney. In conclusion, giant hydronephrosis is an infrequent entity and should be considered in the differential diagnosis of large cystic abdominal masses. The treatment is determined by the underlying cause and the visual appeal of the affected kidney.

## Introduction

Giant hydronephrosis as a result of ureteropelvic junction obstruction; is defined as a dilatation of the pelvicalyceal system comprising more than one liter of urine, causing swelling in the hemiabdomen, or crossing the midline with a cover of five vertebral lines [1]. It is commonly represented on the left side and in men more than in women (male to female ratio: 2.4/1) [2]. Most ureteropelvic junction obstruction cases are discovered during early life or infancy. Even though not treated promptly, it can lead to gradual progression and comorbidities such as hypertension, rupture of affected kidneys, renal impairment, and malignant transformation in some cases [3]. The hydronephrosis range is limited to the pelvicalyceal system in previously reported cases. However, the amount of hydronephrosis in our patient had engaged almost the entire peritoneal cavity (12 liters), and cases such as these are infrequently existing. Thus, we present a case of giant hydronephrosis caused by left ureteropelvic junction obstruction; the diagnosis and treatment options were discussed.

## Patient and observation

**Patient information:** a 16-year-old female patient, illiterate, presented to our outpatient urology clinic in May 2021; with progressive abdominal distention started three months ago and was associated with mild to moderate dull pain and fever. The patient had a history of right renal pelvic stone that underwent double J stent insertion and extracorporeal shock wave lithotripsy five months ago. At that time, the patient family was informed about the left renal ureteropelvic junction obstruction.

**Figure 1:**
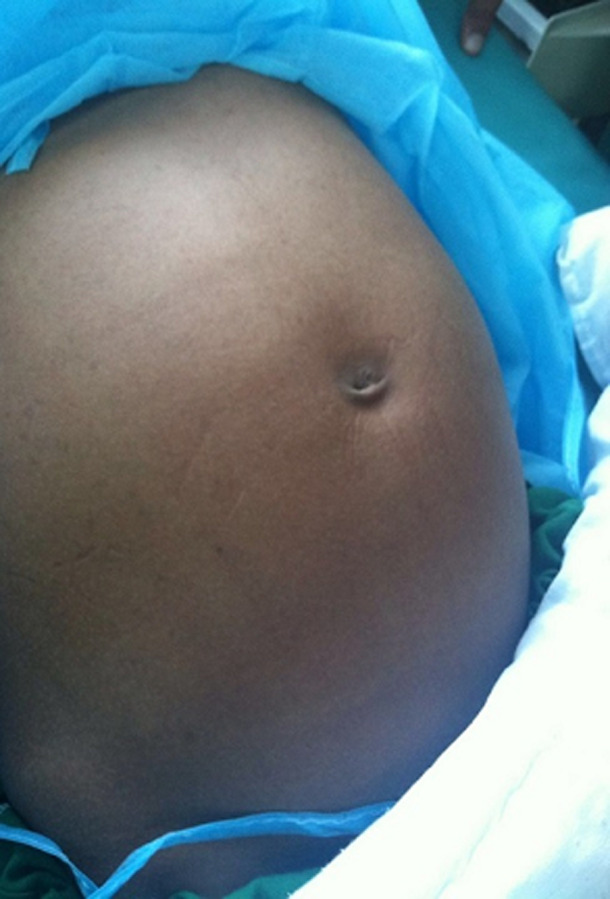
pre-operative photograph showing the abdominal distention

**Clinical findings:** the patients' vital sign was stable (blood pressure 120/70 mmHg, respiratory rate: 14 respirations per minute, pulse rate: 61 beats per minute). The oral temperature was 38.5°C. The total body weight was 52 kg. She was pale, and the abdomen was distended with everted umbilicus and mild abdominal tenderness (Figure 1).

**Figure 2:**
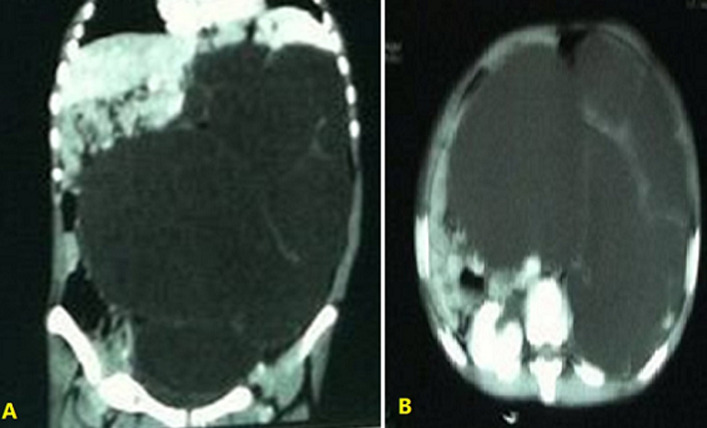
abdominal computed tomography (CT) scan relived giant hydronephrosis of the left kidney: A) coronal views; B) axial views

**Diagnostic assessment:** the white blood cell count of 13x10^3^/ml, hemoglobin: 14.4 g/dl, blood urea nitrogen: 14 mg/dl, and creatinine: 1.1 mg/dl. Urine analysis showed microscopic hematuria and pyuria (15-20 RBCs/HPF and 10-14 WBCs/HPF). Other blood investigation tests, such as liver function tests, were normal. According to her signs and symptoms, ultrasonography was requested that showed massive multicystic lesions of the left kidney with severe hydronephrosis. Abdominal computed tomography scan with intravenous contrast revealed a huge left cystic mass (20x17 cm) with severe hydronephrosis and the renal cortical thickness of about 3 mm and the inferior border of mass reaching the pelvis with no obvious contrast material in the ureter or left renal arteries and caused pressure effects on other peritoneal organs (Figure 2).

**Figure 3:**
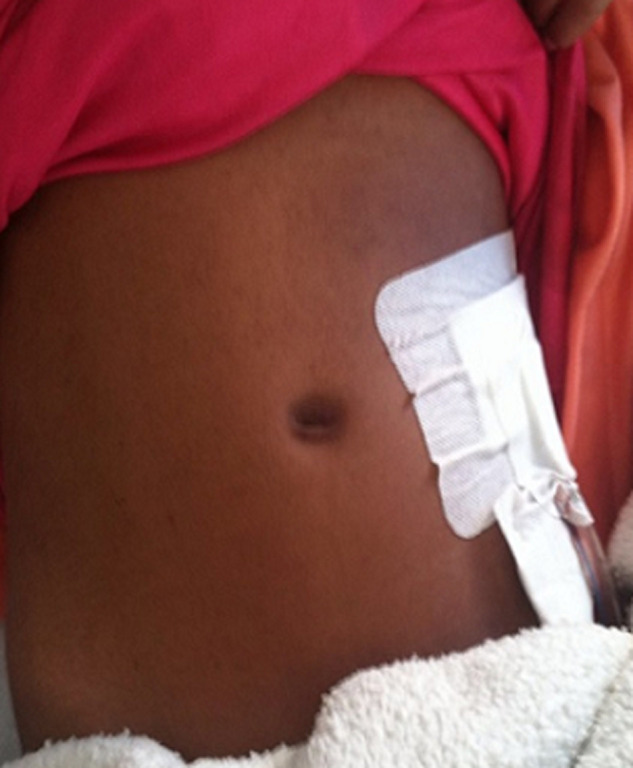
A) the site of surgery after nephrectomy (arrow); B) gross image of the affected kidney with cystic dilatation (arrow)

**Therapeutic interventions:** the patient was admitted as a left ureteropelvic junction obstruction with extremely severe hydronephrotic kidney and prepared for a left nephrectomy. The patient underwent surgical exploration and was approached by a left subcostal incision. Huge retroperitoneal cystic mass was seen from the left flank, crossing the midline to the right side, extending superiorly up to the diaphragm and inferiorly to the pelvis. We could not remove it as one mass due to its enormous size. So, puncturing and aspiration of its content were done, and more than 12 litters of turbid urine were suctioned; after that, a nephrectomy was done (Figure 3). The drain was inserted into the abdominal cavity. Finally, the fascia and skin were closed.

**Figure 4:**
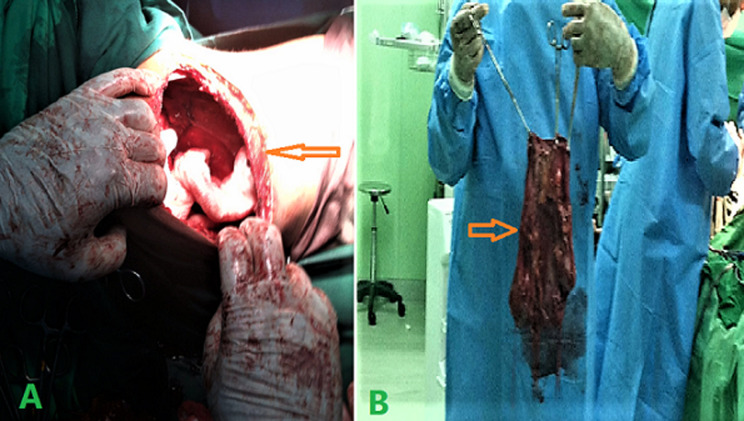
postoperative image of the patient after left nephrectomy

**Follow-up and outcome:** her postoperative recovery was uneventful. The drain was removed on the second postoperative day. The total body weight was 41 kg (lost 11kg) (Figure 4). She was discharged home on the fourth postoperative day, and we advised her family for follow-up for the right solitary kidney. With five-month of follow-ups, no specific complications were observed.

**Patient's perspective:** the patient was happy with the successful outcome of the surgery.

**Informed consent:** a written informed consent was obtained from the patient for participation in our study.

## Discussion

Giant hydronephrosis is an infrequent problem that affects patients of all ages and is described as more than 1000 ml of urine in the pelvicalyceal system. Despite numerous cases of massive hydronephrosis described in the literature, just a few patients presented with more than 2 liters of urine in the pelvicalyceal system [4]. In the current case, the total amount of suctioned urine was more than 12 liters, enough to fill the entire abdominal cavity.

Regarding previous reports, Chiang and associations reported 4 cases of giant hydronephrosis containing between 1.9 to 3.4 litter [5]. Turgut *et al*. reported a case of giant hydronephrosis having five litters of urine in the pelvicalyceal system [6]. Schrader *et al*. described a case of giant hydronephrosis containing more than 15 kg [7]. Additionally, giant hydronephrosis was reported by Yilmaz and associations in a 12 years old boy with 13 liters of urine in the affected kidney [8]. Glass *et al*. reported the giant hydronephrotic kidney in 1746, containing 115 liters of urine [9]. Vilares *et al*. and Para *et al*. reported recent cases of giant hydronephrosis in a 62-year-old man and 19-year-old girl containing 80 liters and 13.5 liters of urine, respectively [10,11]. The most common cause of giant hydronephrosis is ureteropelvic junction obstruction. Other cases are urolithiasis, urinary tract trauma, renal ectopy, and tumor of the ureter [8]. In our case, the left ureteropelvic junction obstruction was the reason for this condition.

These patients' clinical symptoms are not specific, but they commonly include increased abdominal girth and distended abdomen. Other manifestations include flank pain, hematuria, chronic abdominal pain, and repeated urinary tract infections [2]. Even with its vague and general clinical manifestations, only 50% of giant hydronephrosis cases have been correctly diagnosed in the past. However, imaging modalities have advanced significantly over the years. Giant hydronephrosis is defined by ultrasonography as the existence of hydronephrosis that extends further than the abdominal midline or six vertebral bodies with thin renal cortical thickness in radiologic images [12]. Other effective investigative imaging methods are abdominal X-ray, intravenous pyelography, Magnetic resonance imaging, and computerized tomography scan [4,13]. In our case, the diagnosis was made based on clinical symptoms and a computerized tomography scan imaging study. All considered differential diagnoses were abnormal ascites, intrabdominal cysts, adrenal cysts, pseudocysts of the pancreas, and ovarian cysts and tumors [14].

The treatment modality described in published articles includes percutaneous nephrostomy insertion, reconstructive surgery such as ureterocalicostomy, dismembered pyeloplasty, calycovesicostomy, and boari flap with calycovesicostomy, and laparoscopic and open simple nephrectomy. The treatment option is deepened on patient condition, renal functional status, and surgeon experiences [15]. In a patient with loss of renal function, and thin cortical thickness, simple nephrectomy is the optimal choice because of the expected complications such as infections and tumors changes [4]. A puncture/drain procedure such as nephrostomy insertion may be performed if the patients' condition precludes other treatments or if hemodynamic instability occurs due to a sudden abdominal rupture [13]. In our patient, giant hydronephrosis was effectively treated with simple nephrectomy.

## Conclusion

Giant hydronephrosis is a rare condition that must be included in the differential diagnosis of giant abdominal masses. Simple nephrectomy provides a suitable option, such as in our case.

## References

[1] Kamath SP, Ganesh Pai K, Baliga BS (2018). Bilateral giant hydronephrosis in a ten-year-old male. Case Rep Pediatr.

[2] Kaura KS, Kumar M, Sokhal AK, Gupta AK, Purkait B, Saini D (2017). Giant hydronephrosis: still a reality. Turk J Urol.

[3] Boudhaye TI, Sidimalek M, Jdoud C (2017). Giant idiopathic hydronephrosis: toward a two-step therapeutic approach. Pan Afr Med J.

[4] Mediavilla E, Ballestero R, Correas MA, Gutierrez JL (2013). About a case report of giant hydronephrosis. Case Rep Urol.

[5] Chiang PH, Chen MT, Chou YH, Chiang CP, Huang CH, Chien CH (1990). Giant hydronephrosis: report of 4 cases with review of the literature. J Formos Med Assoc.

[6] Yapanoğlu T, Alper F, Özbey İ, Aksoy Y, Demirel A (2007). Giant hydronephrosis mimicking an intraabdominal mass. Turk J Med Sci.

[7] Schrader AJ, Anderer G, von Knobloch R, Heidenreich A, Hofmann R (2003). Giant hydronephrosis mimicking progressive malignancy. BMC Urol.

[8] Yilmaz E, Guney S (2002). Giant hydronephrosis due to ureteropelvic junction obstruction in a child: CT and MR appearances. Clin Imaging.

[9] Dennehy PJ (1953). Giant hydronephrosis in a double kidney. Br J Urol.

[10] Vilares RN, Jesus VM, Talizin TB, Carvalho Dos Anjos Silva G, Cezarino BN, Arap MA (2020). Previously unseen 80L giant hydronephrosis. Urol Case Rep.

[11] Para SA, Wani SA, Murty K (2019). Giant hydronephrotic kidney in adolescence- a rare case report and a review of the literature. Urol Case Rep.

[12] Yang WT, Metreweli C (1995). Giant hydronephrosis in adults: the great mimic. Early diagnosis with ultrasound. Postgrad Med J.

[13] Dino MS, Hassen SM, Tufa TH (2021). Unilateral giant hydronephrosis secondary to ureteropelvic junction obstruction in a middle-aged woman. Case Rep Urol.

[14] Singh NK, Jha B, Khanna R, Khanna NN (1993). Giant hydronephrosis masquerading as massive ascites. Postgrad Med J.

[15] Ansari MS, Danish N, Yadav P, Kaushik VN, Kakoti S, Kumar A (2021). Role of ureterocalicostomy in management of giant hydronephrosis in children in contemporary practice: Indications, outcomes and challenges. J Pediatr Urol.

